# Analysis of platelet and monocyte-to-lymphocyte ratio and diabetes mellitus with benign prostatic enlargement

**DOI:** 10.3389/fimmu.2023.1166265

**Published:** 2023-07-10

**Authors:** Guanheng Chen, Leiguang Feng

**Affiliations:** Department of Laboratory Diagnostics, The First Affiliated Hospital of Harbin Medical University, Harbin, China

**Keywords:** diabetes mellitus, benign prostatic enlargement, benign prostatic hyperplasia, platelet, monocyte-to-lymphocyte ratio, inflammation

## Abstract

**Background:**

The etiology of benign prostatic hyperplasia (BPH) is still elusive. The aim of this study was to provide preventive and prognostic parameters associated with diabetes mellitus with benign prostatic enlargement (BPE).

**Methods:**

Diabetic patients were collected retrospectively from February 2021 to December 2022, including monocyte-to-lymphocyte ratio (MLR). Diabetic patients were divided into two groups by whether the prostate volume was greater than or equal to 30 ml, which were diabetes mellitus without BPE (DM) and diabetes mellitus with BPE (DM+BPE). The baseline characteristics were compared, the risk and protective factors associated with DM+BPE were determined using univariate and multivariate logistic regression, and the parameters associated with prostate volume were determined using correlation analysis.

**Results:**

Of the 671 patients collected, age and prostate volume were significantly higher in the DM+BPE than in the DM; MLR was higher in the DM+BPE than in the DM; and platelet was significantly lower in the DM+BPE than in the DM. Univariate logistic regression showed that age was a risk factor, while protective factors for DM+BPE were lymphocytes and platelet. Multifactorial logistic regression showed that age was a risk factor, while platelet was the protective factor for DM+BPE. In the total overall (n=671), prostate volume was positively correlated with age. Prostate volume was negatively correlated with lymphocytes and platelet. In DM+BPE (n=142), prostate volume was positively correlated with age and MLR.

**Conclusion:**

Platelet was a protective factor for DM+BPE and was negatively correlated with prostate volume, whereas MLR was positively correlated with prostate volume in DM+BPE.

## Introduction

Globally, approximately 537 million adults have diabetes mellitus, 90% of which are type 2, and this number is expected to rise to 783 million by 2045 (1). In China, a research study from 2013 to 2018 showed that the estimated prevalence of diabetes mellitus increased from 10.9% to 12.4% and wasn’t significant improvement in treatment (2). The hyperglycemic state of diabetes mellitus promotes the production of intracellular mitochondrial reactive oxygen species, causing oxidative stress and inflammatory interactions leading to immune dysfunction (3, 4). In addition, it causes diabetes-related macrovascular and microvascular damage due to diabetic hypoxic response (5) as well as chronic hypoxic effects (6), which leads to various complications (7).

Benign prostatic hyperplasia (BPH) is a proliferative prostate gland disease in middle-aged and older men, increasing prostate volume (8). A common symptom is the development of lower urinary tract symptoms (9, 10), and acute urinary retention is a serious complication of BPH (11). It severely reduces the quality of life of middle-aged and older men (12). In recent years, although there has been a slight decline in the number of BPH patients with disability injury and healthy life years, due to the increasing number and advancing age, various urological complications resulting from the enlarged prostate gland have become a high burden worldwide (13, 14). The etiology of BPH is not well understood, but inflammation plays a significant role in it (15). In mouse experiments, five botanicals——Flavonoids, Dihydroartemisinin, Neferine, Curcumin, and Carica papaya leaf extract——have been shown to reduce prostate volume due to their anti-inflammatory properties to alleviate or treat prostate enlargement (16–20). Clinical guidelines state that choosing the appropriate surgical procedure according to the prostate size can reduce complications and is vital to improving patients’ quality of life (21–23). Although hyperglycemia has been reported to increase prostate volume (24, 25), and lymphocytes, free/total prostate-specific antigen (26), neutrophil-to-lymphocyte ratio, platelet-to-lymphocyte ratio, and lymphocyte-to-monocyte ratio have been associated with BPH (27), these did not analyze in detail or separately the relationship between prostate volume and hematological parameters. An analysis of platelet and monocyte-to-lymphocyte ratio and diabetes mellitus with benign prostatic enlargement is not reported in related article.

So far, this is the first article to analyze the relationship between prostate volume and hematological parameters in diabetic patients. Therefore, we performed this clinical study to provide clinicians with aid in judgment and to provide new ideas to study the etiology of BPH.

## Patients and methods

### Study population

Diabetic patients were collected retrospectively from February 2021 to December 2022 in the Department of Endocrinology with diabetes mellitus from a query on the laboratory information system at the First Affiliated Hospital of Harbin Medical University.

### Data collection

The patients’ age, fasting blood glucose (FBG), glycated hemoglobin A1c (HbA1c), high-density lipoprotein (HDL), neutrophils, lymphocytes, monocytes, and platelet were recorded, and the patients’ anterior-posterior diameter, upper-lower diameter, and left-right diameter of the prostate were examined by abdominal ultrasound.

### Calculation of MLR, LHR, and prostate volume

MLR= monocytes/lymphocytes (MLR), LHR = lymphocytes/high-density lipoprotein, and prostate volume (PV) = 0.52 × anterior-posterior diameter × upper-lower diameter × left-right diameter.

### Patient selection

We set inclusion criteria and exclusion criteria. Inclusion criteria :(1) males older than or equal to 40 years of age;(2) diabetes mellitus diagnosis;(3) complete test data; and (4) no serious infectious diseases. Exclusion criteria:(1) diabetes mellitus complicated by eye disease;(2) acute complications of diabetes mellitus, such as diabetic ketoacidosis and hyperosmolar hyperglycemic syndrome;(3) diagnosis of prostate cancer;(4) history of prostatectomy; and (5) history of hepatitis.

### Study grouping

The collected diabetic group was divided into two groups according to whether the prostate volume was more significant than or equal to 30 ml, which were divided into the group with diabetes mellitus without benign prostatic enlargement (DM) and the group with diabetes mellitus with benign prostatic enlargement (DM+BPE).

### Statistical analysis

SPSS 25.0 (SPSS, Inc., Chicago, IL, USA) was used for statistical analyses. All measures were tested for normality by the Kolmogorov-Smirnov normality test, and the mean ± standard deviation (SD) was used for data that conformed to a normal distribution. The median and quartiles IQR(Q3-Q1) was used for data that did not work to a normal distribution. If the chi-square test was satisfied between the two groups, the two independent samples t-test was used for the means of the two samples, and when data didn’t conform to normal distribution or didn’t satisfy the chi-square, the two independent samples’ Mann-Whitney U test was used for comparison between the two groups. The binary logistic regression model was established with DM+BPE assigned to 0 and DM set to 1. Univariate logistic regression was performed first, after which statistically significant ones were included in the multifactorial logistic regression to control for confounding factors.

Both variables were normally distributed using Pearson for linear correlation analysis, and didn’t meet normal distribution using Spearman for linear correlation analysis. A two-sided test was used, and *P*<0.05 was considered statistically significant.

## Results

A total of 671 patients were collected. In the comparison between DM and DM+BPE, age 64.00 [57.75-69.00] was significantly higher in the DM+BPE than in the DM 55.00 [48.50-61.50] (*P*<0.001); prostate volume 39.96 [34.31-51.62] was significantly higher in DM+BPE than in DM 20.37 [17.22 -24.13] (*P*<0.001); and MLR 0.24 [0.19-0.31] was higher in the DM+BPE than in the DM 0.22 [0.17-0.28] (*P*=0.029). Lymphocytes 1.82 [1.50-2.21] were lower in the DM+BPE than in the DM 1.97 [1.58-2.41] (*P*=0.011); platelet 201.63 ± 44.68 was significantly lower in the DM+BPE than in the DM 218.36 ± 52.64 (*P*<0.001); and LHR 1.84 [1.40-2.28] was lower in the DM+BPE was lower than in the DM 1.91 [1.45-2.57] (*P*=0.040). All other clinical parameters were not statistically significant (*P*>0.05), and the results were shown in **Table 1**.

**Table 1:** Baseline characteristics of diabetes mellitus with or without benign prostatic enlargement.

Variables	Overall(n=671)	DM(n=529)	DM+BPE(n=142)	Z/t	*P* Value
Age, years, median (IQR)	57.00[50.00-63.00]	55.00[48.50-61.50]	64.00[57.75-69.00]	-9.021	<0.001
FBG, mmol/L, median (IQR)	8.11[6.48-10.19]	8.21[6.50-10.21]	7.84[6.35-10.07]	0.993	0.321
HbA1c, %, median (IQR)	8.30[7.10-9.60]	8.40[7.20-9.65]	8.00[6.90-9.43]	1.878	0.060
HDL, mmol/L, median (IQR)	1.01[0.88-1.18]	1.00[0.88-1.17]	1.01[0.88-1.20]	-0.133	0.894
Leukocytes,10^9^/L, median (IQR)	6.32[5.42-7.46]	6.37[5.53-7.53]	6.15[5.10-7.24]	1.730	0.084
Neutrophils,10^9^/L, median (IQR)	3.70[2.93-4.60]	3.72[2.95-4.61]	3.61[2.86-4.47]	0.767	0.443
Lymphocytes,10^9^/L, median (IQR)	1.95[1.56-2.37]	1.97[1.58-2.41]	1.82[1.50-2.21]	2.530	0.011
Monocytes,10^9^/L, median (IQR)	0.43[0.35-0.53]	0.44[0.35-0.53]	0.43[0.36-0.53]	-0.192	0.848
Platelet,10^9^/L, mean (SD)	214.82 ± 51.48	218.36 ± 52.64	201.63 ± 44.68	-3.809	<0.001
PV, ml, median (IQR)	22.14[18.05-28.64]	20.37[17.22-24.13]	39.96[34.31-51.62]	-18.313	<0.001
MLR, median (IQR)	0.23[0.18-0.28]	0.22[0.17-0.28]	0.24[0.19-0.31]	-2.187	0.029
LHR, median (IQR)	1.89[1.45-2.53]	1.91[1.45-2.57]	1.84[1.40-2.28]	2.058	0.040

DM, diabetes mellitus without benign prostatic enlargement group; DM+BPE, diabetes mellitus with benign prostatic enlargement group; FBG, fasting blood glucose; HbA1c, glycated hemoglobin A1c; HDL, high-density lipoprotein; PV, prostate volume; MLR, monocyte-to-lymphocyte ratio; LHR, lymphocyte-to-high-density lipoprotein ratio.

A univariate logistic regression analysis of age, FBG, HbA1c, HDL, leukocytes, neutrophils, lymphocytes, monocytes, platelet, MLR, and LHR, which were parameters associated with DM+BPE, showed that the risk factor for DM+BPE was age (OR=1.113, 95% CI=1.086~1.140, *P*<0.001), while the protective factors for DM+BPE were lymphocytes (OR=0.657, 95% CI=0.482~0.896, *P*=0.008); platelet (OR=0.093, 95% CI=0.990~0.997, *P*=0.001); and LHR (OR=0.742, 95% CI=0.582~0.947, *P*=0.016). All other clinical parameters were not statistically significant (*P*>0.05), and the results were manifested in **Table 2**.

**Table 2:** Univariate logistic regression analysis of diabetes mellitus with and without benign prostatic enlargement.

Variables	β	SE	Wald χ^2^	*P* Value	OR	95% CI
Age, years	0.107	0.012	75.436	<0.001	1.113	1.086~1.140
FBG, mmol/L	-0.039	0.033	1.344	0.246	0.962	0.901~1.027
HbA1c, %	-0.094	0.054	3.106	0.078	0.910	0.819~1.011
HDL, mmol/L	-0.120	0.308	0.152	0.696	0.887	0.485~1.621
Leukocytes, 10^9^/L	-0.104	0.056	3.405	0.065	0.901	0.807~1.007
Neutrophils, 10^9^/L	-0.063	0.068	0.865	0.352	0.939	0.822~1.072
Lymphocytes,10^9^/L	-0.420	0.158	7.062	0.008	0.657	0.482~0.896
Monocytes, 10^9^/L	-0.205	0.478	0.184	0.668	0.815	0.320~2.077
Platelet, 10^9^/L	-0.007	0.002	11.623	0.001	0.093	0.990~0.997
MLR	0.009	0.232	0.002	0.968	1.009	0.641~1.590
LHR	-0.298	0.124	5.766	0.016	0.742	0.582~0.947

FBG, fasting blood glucose; HbA1c, glycated hemoglobin A1c; HDL, high-density lipoprotein; MLR, monocyte-to-lymphocyte ratio; LHR, lymphocyte-to-high-density lipoprotein ratio.

Age, lymphocytes, platelet, and LHR were statistically significant in univariate logistic regression. Considering age as a confounding factor for BPH, we performed multifactorial logistic regression analysis for the above parameters. The results showed that the risk factor for DM+BPE was age (OR=1.109, 95% CI=1.082~1.137, *P*<0.001), and the protective factor for DM+BPE was platelet (OR=0.995, 95% CI=0.991~0.999, *P*=0.017). All other clinical parameters were not statistically significant (*P*>0.05), and the results were exhibited in **Table 3**.

**Table 3:** Multifactorial logistic regression analysis of diabetes mellitus with and without benign prostatic enlargement.

Variables	β	SE	Wald χ^2^	*P* Value	OR	95% CI
Age, years	0.104	0.013	68.323	<0.001	1.109	1.082~1.137
Lymphocytes,10^9^/L	-0.076	0.215	0.123	0.725	0.927	0.608~1.414
Platelet,10^9^/L	-0.005	0.002	5.696	0.017	0.995	0.991~0.999
LHR	0.015	0.204	0.006	0.940	1.015	0.680~1.516

LHR, lymphocyte-to-high-density lipoprotein ratio.

In the correlation analysis, we subjected the total overall (n=671) to Spearman correlation analysis, and the positive correlation identified in this analysis was prostate volume with age (r=0.309, *P*<0.001). The negative correlations were prostate volume with lymphocytes (r=-0.091, *P*=0.018) and platelet (r=-0.098, *P*=0.011). In the DM+BPE (n=142) Spearman correlation analysis, the positive correlations identified in this analysis were prostate volume with age (r=0.182, *P*=0.030), MLR (r=0.236, *P*=0.005), and no other clinical parameters were statistically significant (*P*>0.05), as demonstrated in **Table 4**.

**Table 4:** Correlation between prostate volume and other parameters.

Overall(n=671)	PV, ml		DM+BPE(n=142)	PV, ml	
	r Value	*P* Value		r Value	*P* Value
Age, years	0.309	<0.001		0.182	0.030
FBG, mmol/L	-0.045	0.245		0.001	0.986
HbA1c, %	-0.033	0.398		-0.055	0.515
HDL, mmol/L	-0.041	0.287		-0.177	0.166
Leukocytes,10^9^/L	-0.063	0.102		0.097	0.249
Neutrophils,10^9^/L	-0.034	0.381		0.141	0.094
Lymphocytes,10^9^/L	-0.091	0.018		-0.162	0.053
Monocytes,10^9^/L	-0.019	0.620		0.114	0.176
Platelet,10^9^/L	-0.098	0.011		0.104	0.220
MLR	0.059	0.128		0.236	0.005
LHR	-0.054	0.161		-0.048	0.570

PV, prostate volume; DM+BPE, diabetes mellitus with benign prostatic enlargement group; FBG, fasting blood glucose; HbA1c, glycated hemoglobin A1c; HDL, high-density lipoprotein; MLR, monocyte-to-lymphocyte ratio; LHR, lymphocyte-to-high-density lipoprotein ratio.

## Discussion

The hyperglycemic state of diabetes mellitus may affect BPH (28). The systemic chronic low-grade inflammation of diabetes mellitus promotes the development of prostatic enlargement (29). The inflammation increases the size of the prostate (30), which leads to bladder outlet obstruction (31). It severely reduces sleep quality in middle-aged and older men (32) and sometimes causes depression (33). Lower urinary tract symptoms are caused by prostatic enlargement blocking the bladder outlet (34). If not properly managed, it causes the development of urinary tract infections (35, 36). Increasing age is one of the risk factors for BPH (37). The increase in prostate volume with increasing age in our present analysis is consistent with previous reports in the literature (38). This confounding factor must be controlled for in our current study.

Lymphocytes have a scavenging effect on foreign antigens, and the LHR reflects the body’s inflammatory status. Increased HDL is negatively associated with the risk of developing BPH (39). Lipid-lowering drugs have a therapeutic effect on BPH (40). Thus, in this study, lymphocytes and LHR levels were reduced in **Table 1** but not statistically significant in the analysis of **Tables 2**, **3**, probably because age was among the confounding factors. Platelet and MLR parameters have been shown to have a prognostic role in idiopathic pulmonary fibrosis (41), acute ischemic stroke (42), and immunoglobulin A nephropathy (43). However, there need to be more reports on DM+BPE. In our study, platelet was a protective factor for BPE, and prostate volume was negatively correlated with platelet counts. Some substances secreted by platelet may have an imbalance that positively regulates cell proliferation in the prostate. Although MLR wasn’t statistically significant in **Tables 2**, **3**, in **Tables 1**, **4**, there was a positive correlation between prostate volume and MLR in DM+BPE, probably due to an inflammatory response caused by monocyte infiltration leading to an increase in prostate volume (44, 45).

Our study has some advantages: On the one hand, this study provides valuable parameters for prevention, treatment, and determination of prognosis valid for patients with DM+BPE. On the other hand, because platelet and MLR are readily available in the physical blood test, they have a relatively low price and are more suitable for widespread use. However, some shortcomings must be addressed: Firstly, because this was a retrospective study, data were collected without specimens corresponding to all patients, lacking the indicator of prostate-specific antigen, which is associated with prostate volume (46, 47). An increased prostate-specific antigen concentration is a risk for enlarged prostate volume (48). Secondly, this single-center clinical study may influence ethnicity, geography, and social and economic conditions. Finally, this study could not prove causality because it was retrospective. Therefore, prostate-specific antigen parameters should be increased in the future, and prospective and multicenter studies should be conducted to provide evidence-based surgical treatment for patients with BPH.

## Conclusion

Platelet was a protective factor for DM+BPE and was negatively correlated with prostate volume in the overall patient analysis. In DM+BPE, MLR was positively correlated with prostate volume.

## Data availability statement

The original contributions presented in the study are included in the article/supplementary material. Further inquiries can be directed to the corresponding author.

## Ethics statement

The studies involving human participants were reviewed and approved by the Ethics Committee of the First Affiliated Hospital of Harbin Medical University (202247). The patients/participants provided their written informed consent to participate in this study.

## Author contributions

GC Data acquisition, Data analysis, and Writing. LF Funding acquisition, Conceptualization, and Supervision. Both authors contributed to the article and approved the submitted version.
